# Contribution to the Prediction of the Fold Code: Application to Immunoglobulin and Flavodoxin Cases

**DOI:** 10.1371/journal.pone.0125098

**Published:** 2015-04-27

**Authors:** Mateusz Banach, Nicolas Prudhomme, Mathilde Carpentier, Elodie Duprat, Nikolaos Papandreou, Barbara Kalinowska, Jacques Chomilier, Irena Roterman

**Affiliations:** 1 Department of Bioinformatics and Telemedicine, Medical College, Jagiellonian University, Krakow, Poland; 2 Protein Structure Prediction group, IMPMC, UPMC & CNRS, Paris, France; 3 RPBS, 35 rue Hélène Brion, 75013, Paris, France; 4 Genetics Department, Agricultural University of Athens, Iera Odos 75, Athens, Greece; Indian Institute of Science, INDIA

## Abstract

**Background:**

Folding nucleus of globular proteins formation starts by the mutual interaction of a group of hydrophobic amino acids whose close contacts allow subsequent formation and stability of the 3D structure. These early steps can be predicted by simulation of the folding process through a Monte Carlo (MC) coarse grain model in a discrete space. We previously defined MIRs (Most Interacting Residues), as the set of residues presenting a large number of non-covalent neighbour interactions during such simulation. MIRs are good candidates to define the minimal number of residues giving rise to a given fold instead of another one, although their proportion is rather high, typically [15-20]% of the sequences. Having in mind experiments with two sequences of very high levels of sequence identity (up to 90%) but different folds, we combined the MIR method, which takes sequence as single input, with the “fuzzy oil drop” (FOD) model that requires a 3D structure, in order to estimate the residues coding for the fold. FOD assumes that a globular protein follows an idealised 3D Gaussian distribution of hydrophobicity density, with the maximum in the centre and minima at the surface of the “drop”. If the actual local density of hydrophobicity around a given amino acid is as high as the ideal one, then this amino acid is assigned to the core of the globular protein, and it is assumed to follow the FOD model. Therefore one obtains a distribution of the amino acids of a protein according to their agreement or rejection with the FOD model.

**Results:**

We compared and combined MIR and FOD methods to define the minimal nucleus, or keystone, of two populated folds: immunoglobulin-like (Ig) and flavodoxins (Flav). The combination of these two approaches defines some positions both predicted as a MIR and assigned as accordant with the FOD model. It is shown here that for these two folds, the intersection of the predicted sets of residues significantly differs from random selection. It reduces the number of selected residues by each individual method and allows a reasonable agreement with experimentally determined key residues coding for the particular fold. In addition, the intersection of the two methods significantly increases the specificity of the prediction, providing a robust set of residues that constitute the folding nucleus.

## Introduction

As it has been enunciated by Oleg Ptitsyn in the early 90s, “the code of crude protein 3D structure is highly degenerate, i.e. quite different sequences can have similar tertiary folds, thus emphasizing that not all details of sequence, (i.e. not all interactions) are important for this coding” [1]. In line with this statement, the present work is an integration of two approaches, in order to better decipher some key residues that can be considered as a signature of a given globular protein fold. There are several expectations in predicting such a signature. Kister & Gelfand showed that a limited number of key residues determine the super secondary structures [2] and, if one has a sufficient number of intra chain contacts, the type of fold can then be predicted, according to Jones et al. [3]. It also improves the quality of the predicted models [4] or it may be a necessary condition to start a model with rigid secondary structures [5]. Besides, this knowledge can give insights into kinetics, since the distribution and strength of the contacts in the native structure largely determine the folding speed [6]. Prediction of intra chain residue contacts has then been introduced as a category in the CASP competition (Critical Assessment of techniques for protein Structure Prediction, at http://predictioncenter.org), because it allows either filtering the models generated by threading algorithms, or reducing the conformational space in molecular modelling [7]. It has been suggested that, for small proteins, just one correctly predicted contact for every seven residues would be enough to build approximate models [8].

**Fig 1:**
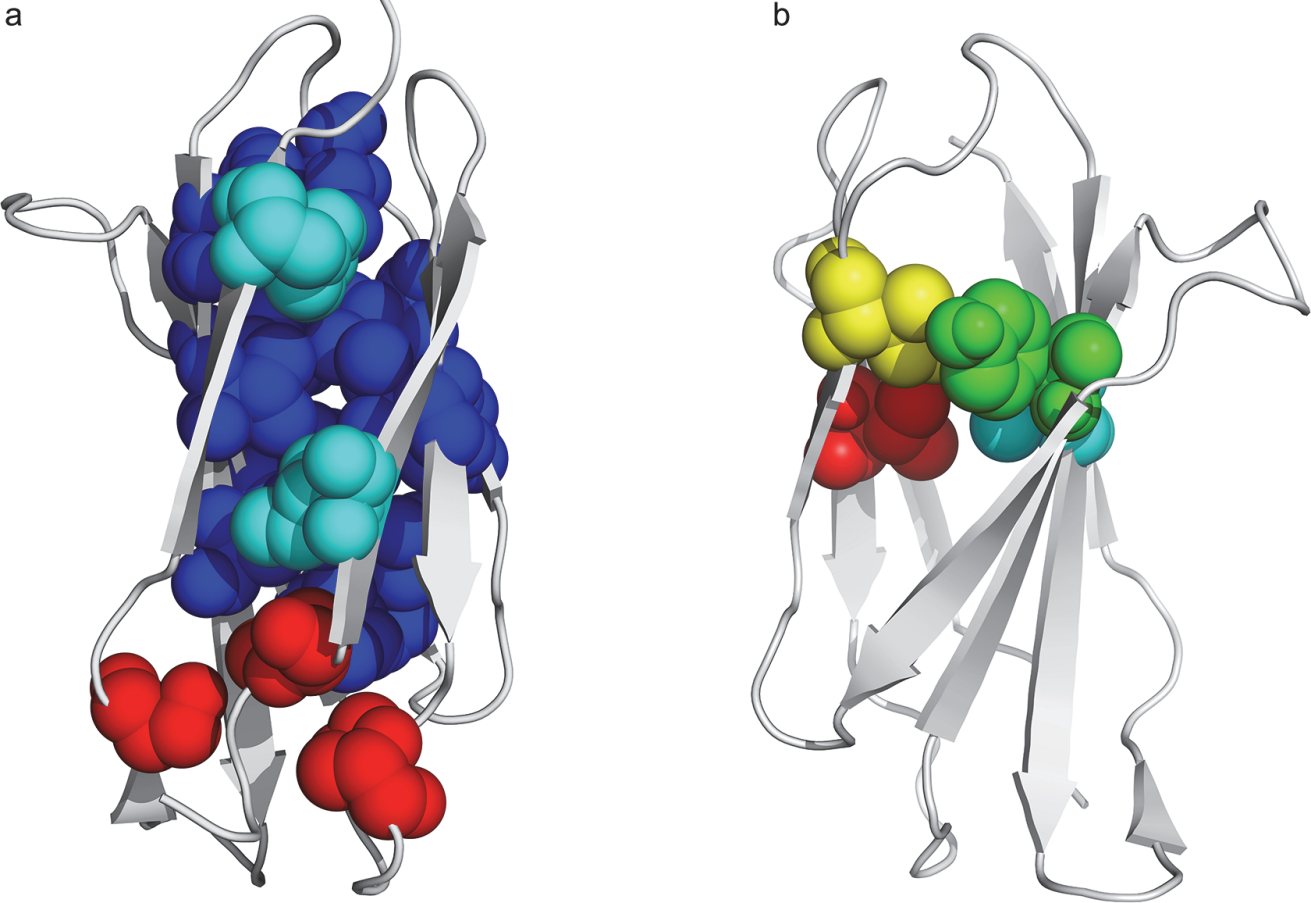
Difference from core and folding nucleus of the immunoglobulin-like fold, redrawn from results established by the group of Jane Clarke. A) The core corresponds to the CATH code domain 2ck2A00 [28]; in red peripheral core residues, in cyan I8 and F92 and in blue the rest of the core. B) The nucleus according to Clarke’s numbering (I20 in red, Y36 in green, I59 in yellow and V70 in cyan) for the CATH code domain 1tenA00 [19].

Amino acids involved in a high number of intra chain contacts are generally buried in the interior of the globular proteins. One must make here some comments on the vocabulary, to distinguish between core and nucleus, as it has already been proposed [9]. In the literature, the concept of core is usually employed to analyse the structure of a globular protein: it contains all the residues not exposed to the solvent, therefore mainly hydrophobic, but not exclusively. Following Rose and Cramer, the core “is comprised of residues that are distant in sequence but close in space, suggesting a comprehensive architectural plan” [10]. Determination of a hydrophobic core in globular proteins is not trivial. Neither solvent accessibility, nor mapping of amino acids according to some hydrophobicity scale, are sufficient to accurately determine the set of residues necessary for the formation and stabilization of the tertiary structure. Different approaches have been developed to tackle this issue [11–22]. Each one has qualities and defaults, but the identity or hydrophobicity of residues are poor tools for extracting information from protein cores, rendering methods based only on sequence alignments unsatisfactory [23]. The concept of folding nucleus refers to a set of amino acids, mainly hydrophobic, interacting during the transition states of the folding process, and eventually dispersed along the sequence. The interactions of these residues, due to the thermal vibrations of the chain, are required for the protein to obtain its native structure. Originally proposed by Ptitsyn, the nucleus concept has been linked to the condensation nucleation model of folding [24,25]. It has also been proposed as a more general transition state [26], either diffuse or polar. These transient interactions do not necessarily remain in the native structure. The methods generally employed so far for nucleus prediction are multiple alignments (through the identification of the evolutionary conserved hydrophobic residues), molecular dynamics of the unfolding, SVM [7] or exhaustive enumeration of the transition pathways [27]. To sum up, globular core is a static concept, while folding nucleus is a dynamical one. One can visualize an example of the difference when comparing in Fig 1 the core [28] and the nucleus [19] in the case of an immunoglobulin-like fold domain (fibronectin type III). As far as this paper is concerned, we will consider the folding nucleus as the minimal number of amino acids, interacting in the first steps of the folding reaction. They remain in close contacts in the native structure, so it justifies the cumulative approach described here, a simulation of the folding and an analysis of the actual final structure.

In this paper, the methods developed by our two groups, namely MIR (Most Interacting Residue) approach on one side, and FOD approach on the other side, are compared and combined in order to increase the precision on identification of the key residues coding for a given fold. The MIR method is an *ab initio* simulation of the protein folding, starting from the sequence as unique input [29,30]. It calculates the number of non-covalent neighbours of each amino acid in a 3D lattice and it assigns the maxima of this distribution as Most Interacting Residues (MIR). MIRs are therefore mainly buried and assumed to be related to the folding nucleus in the case of the immunoglobulin fold [31]. Depending on the protein domain, the proportion of MIR is in the range [15–20]% of its sequence length, while in the experimental literature, the range for the folding nucleus is generally [5–10]% although one can find much larger values [32], up to 60% [27,33].

In the second approach, the FOD method, the hydrophobic core is analysed from the input of a 3D structure to detect structural similarities within observed and theoretical hydrophobicity distributions. In order to predict whether a given residue belongs or not to the hydrophobic core, the “fuzzy oil drop” (FOD) model [25,34–36] takes as a criterion the discrepancy between expected (let us call it idealized) and observed hydrophobicity density profiles. It goes beyond the simple use of a null solvent accessibility of amino acids as a criterion to determine the hydrophobic core. The output is a set of residues accordant with the FOD model, i.e. for which idealized and experimental hydrophobic densities are either both high or both low. The key point is the agreement between theory and observation.

In short, MIR deals with the folding nucleus prediction from the sequence, and FOD deals with the core determination from the structure. Benefits and drawbacks of respective methods can be balanced if one concentrates on the determination of the set of amino acids one can consider as a signature of a fold, in line with the “fold code” concept proposed by Rose and Creamer: what is the minimum number of amino acids necessary to produce one fold instead of another one [10]. This gave rise to the famous Paracelsus challenge awarding 1,000 dollars to the group that would successfully change the fold of one protein by changing no more than half the sequence. We then expect to import, from the analysis of the structures by the FOD method, pertinent rules that will be used as input in the MIR algorithm, which will hopefully decrease the number of false positive positions predicted by the MIR method. This is why we will focus on the early steps of the folding process. Besides, this goal allows escaping from the controversy about the conservation of the folding nucleus among the proteins of a given fold, with an expected high conservation on one hand [14,37] or its absence on the other hand [38].

Two protein domain datasets have been explored in this analysis. Actually, immunoglobulin-like (Ig) fold is not a protein family, or super family, in strict evolutionary terms, but is one of ten proposed “superfolds” adopted by unrelated sequences [39]. It is widely admitted that its folding occurs through a nucleation condensation mechanism, therefore with the formation of a nucleus in the transition state. In immunoglobulin-like domains, “functional and structural load is clearly separated: loops are responsible for binding and recognition while interactions between several residues of the buried core provide stability and fast folding” [14]. Sharing this fold, 56 domains are considered, presenting highly divergent sequences, with a mean pair sequence identity of 9.5%, and a large variety of functions. PFAM alignment in the immunoglobulin superfamily confirms that the core is highly conserved, while it has no fix position for the type III fibronectin, as far as one considers the examples of TNf3 and CAfn2, but transition states are assumed conserved when considering Phi value analysis, indicating some similarity in the folding pathways among the two super families [40]. The second dataset, the flavodoxin (Flav) fold, which also occurs via a nucleation mechanism [41], contains 38 domains of divergent sequences with a mean pair sequence identity of 6.9%. One of the reasons of this choice is because it belongs to the five most common observed folds, with a broad range of unrelated proteins with different functions [42]. Its folding process goes through intermediate states, either on or off pathway, therefore the mechanism is not shared among all members of this fold [43]. The fact the transition state ensemble of some of the proteins sharing this fold is diffuse (an expanded form of the folded state) [44] may not, a priori, necessarily lead to the conservation of the highly interacting residues. Besides, since some of the residues with high Phi values are not hydrophobic [45], this fold is worth investigating the conservation of the key residues.

The combination of these two methods is expected to improve the accuracy of the prediction. Consideration of a set of proteins instead of a single sequence should allow to extract the conserved portion of the nucleus, giving insights into the set of amino acids compulsory to dictate the type of fold in which these proteins will find their native structure, what we can call the fold coding residues.

## Material and Methods

### Datasets

One of the most frequent folds is the immunoglobulin beta-sandwich. Among this large group of proteins, there is no shared function, and sequence similarities can go down to very low values although there is a common structural core [46]. Proteins used in a former analysis [30] were taken as the current data set and we added four immunoglobulin domains with experimental data related to their folding nucleus. A multiple sequence alignment was produced for the immunoglobulin fold, by adding their sequences to the former one, according to the pairwise best-hit structural superimpositions generated using DALI [47]. The multiple sequence alignment of flavodoxin domains was derived from the superimposition of their structures computed by MUSTANG [48]. The list of domains with short characteristics is given in Table 1 of the paper by Prudhomme and Chomilier [31] for the immunoglobulin-like fold, and in Table 1 of this paper for the flavodoxin fold. The data set includes 94 single-segment domains: 56 immunoglobulin-like and 38 flavodoxins, whose limits have been retrieved from the CATH database [49]. Mean length of the domains is 108 (standard deviation 19) for the immunoglobulin-like fold and 171 (standard deviation 39) for the flavodoxin fold. Precision on the limits of the domains could affect the FOD assignment if some significant length, typically one sheet (at least 5 amino acids) oriented outside of the globule, is added or removed. This is not a major concern in our study. Concerning MIR, there are some side effects that decrease the reliability of the prediction at both N and C terminals, but apart the ends where the results must be taken with care, the precision can be estimated at ±1 amino acids in the bulk of the sequence.

**Table 1:** Description of the 38 domains with a flavodoxin fold used as a dataset.

Protein	Origin	PDB	Res	Chain	Domain
Glutamate mutase	*Clostridium tetanomorphum*	1BE1	NMR	A	00(1–137)
B-12-binding domains of methionine synthase	*Escherichia coli*	1BMT	3	A	02(738–895)
D-2-hydroxyisocaproate dehydrogenase	*Lactobacillus casei*	1DXY	1.86	A	02(102–297)
CheY (signal transduction protein)	*Escherichia coli*	1EAY	2	A	00(2–129)
Serine esterase	*Streptomyces scabiei*	1ESD	2.3	A	00(4–305)
GTP-specific succynil-CoA synthetase	*Sus scrofa*	1EUC	2.1	A	01(9–131)
	*Sus scrofa*	1EUC	2.1	A	02(132–301)
	*Sus scrofa*	1EUC	2.1	B	03(247–392)
Nucleoside 2-deoxyribosyl transferase	*Lactobacillus leichmannii*	1F8Y	2.4	A	00(2–157)
Apo flavodoxin	*Nostoc sp*	1FTG	2	A	00(2–169)
Platelet-activating factor acetylhydrolase IB	*Bos taurus*	1FXW	2.1	A	00(5–215)
	*Bos taurus*	1FXW	2.1	F	00(6–217)
Tir domain of human TLR1	*Homo sapiens*	1FYV	2.9	A	00(625–785)
NAD-dependent D-glycerate dehydrogenase	*Hyphomicrobium methylovorum*	1GDH	2.4	A	02(102–285)
Type II dehydroquinase	*Bacillus subtilis*	1GQO	2.1	A	00(1–141)
Type II dehydroquinase	*Streptomyces coelicolor*	1GTZ	1.6	A	00(2–150)
Precorrin-8X methylmutase	*Pseudomonas denitrificans*	1I1H	2.6	A	00(2–210)
D-lactase dehydrogenase	*Lactobacillus delbrueckii subsp bulgaricus*	1J4A	1.9	A	02(104–299)
Succinyl-CoA synthetase	*Escherichia coli*	1JKJ	2.35	A	01(1–122)
	*Escherichia coli*	1JKJ	2.35	A	02(123–287)
Lysophospholiase L1/acyl-CoA thioesterase I/proteaseI	*Escherichia coli*	1JRL	1.95	A	00(1–179)
S-adenosylhomocysteine hydrolase	*Homo sapiens*	1LI4	2.01	A	02(193–352)
CtBP dehydrogenase	*Homo sapiens*	1MX3	1.95	A	02(125–318)
Phosphoribosylalinoimidazole mutase	*Thermotoga maritima*	1O4V	1.77	A	00(2–170)
Succinyl-CoA synthetase	*Thermus thermophilus*	1OI7	1.23	A	01(1–122)
PurE (lyase)	*Escherichia coli*	1QCZ	1.5	A	00(7–169)
L-alanine dehydrogenase	*Phormidium lapideum*	1SAY	2.1	A	02(129–304)
PurE (N5-carboxyaminoimidazole ribonucleotide mutase)	*Acetobacter aceti*	1U11	1.55	A	00(20–178)
Type II 3-dehydroquinate dehydratase	*Actinobacillus pleuropneumoniae*	1UQR	1.7	A	00(1–146)
S-adenosyl-I-homocysteine hydrolase	*Plasmodium falciparum*	1V8B	2.4	A	02(237–397)
PurE (lyase)	*Bacillus anthracis*	1XMP	1.8	A	00(2–156)
D-3-phosphoglycerate dehydrogenase	*Mycobacterium tuberculosis*	1YGY	2.3	A	02(99–280)
Lipase / acylhydrolase	*Enterococcus faecalis*	1YZF	1.9	A	00(1–195)
GDSL-like lipase	*Nostoc sp*. *PCC 7120*	1Z8H	2.02	A	00(5–206)
Acetylxylan esterase	*Clostridium acetobutylicum*	1ZMB	2.61	A	01(2–248)
Product of gene AT4G34215	*Arabidopsis thaliana*	2APJ	1.6	A	00(17–260)
H.pylori type II dehydroquinase	*Helictobacter pylori*	2C4V	2.5	A	00(1–158)
Methylmalonyl-CoA mutase	*Propionibacterium freudenreichii subsp*. *shermanii*	7REQ	2.2	A	02(563–726)

Res is the resolution in ångström for X-Ray structures, otherwise they provide from NMR. Limits in domain column are retrieved from CATH. Proteins are sorted according to their PDB code.

### Intra chain contact prediction by the MIR method

**Fig 2:**
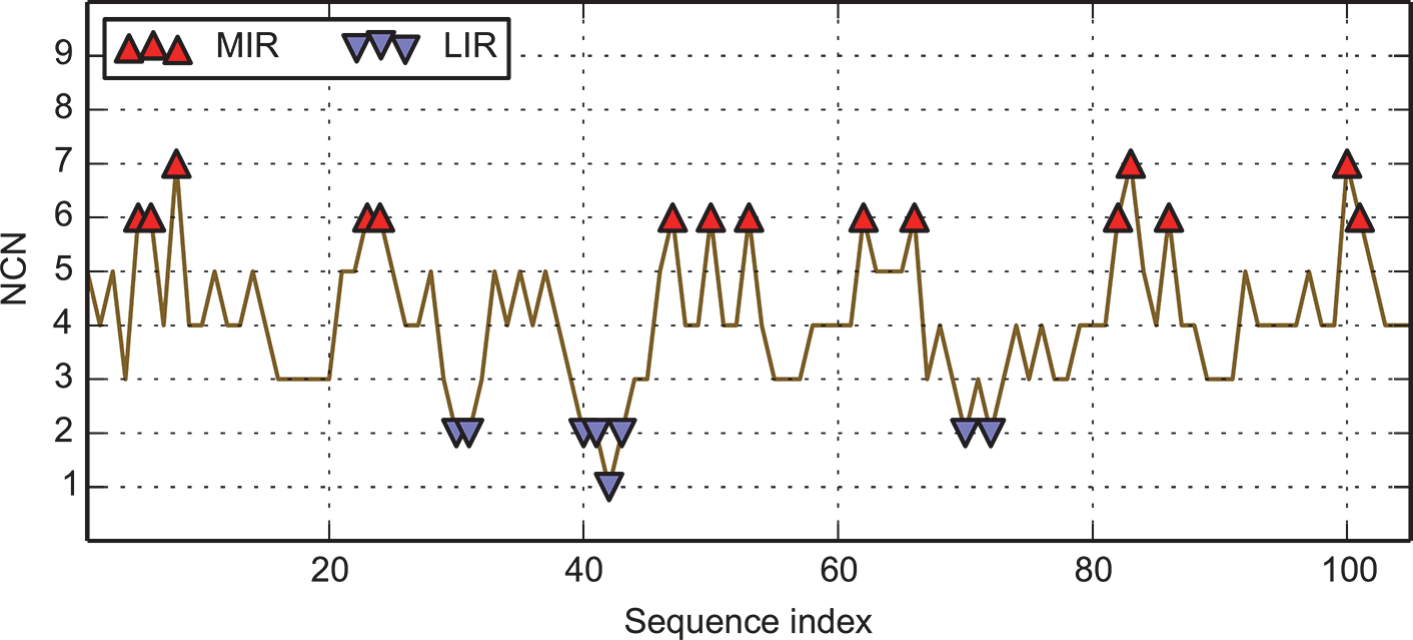
Mean number of Non Covalent Neighbours (NCN) determination for the immunoglobulin-like fold domain 1svcP02. The ordinate is the number of non-covalent neighbours, NCN, and the abscissa is the sequence. Values of the mean NCN above a given threshold of 6 are MIR and values below a given threshold of 2 are LIR. They are indicated by red and blue triangles, respectively.

MIR method is extensively described in [29,30,50] and is briefly summarised here. Starting from an initial conformation of the protein chain with the amino acids randomly placed at the free nodes of a (2,1,0) lattice, one occupied node is chosen by chance at each step of the procedure and is moved to an empty neighbouring node. Compared to a simple cubic lattice, this lattice proposed by Kolinski et al. [51] has two advantages: 1) it allows angles between three successive alpha carbons ranging from 66° and 143°; 2) the number of first neighbours increases from 6 to 24. Reducing each amino acid to a single alpha carbon allows a huge computer time reduction, and it is coherent with the coarse grain model proposed in this study, limited to the early steps of the folding process. If explicit inclusion of the side chain were realised, it would only improve the results in the fine details of the folding [52], but not in the early steps of this process since partial chain packing occurs gradually [53]. The energies of the initial and final conformations are calculated by means of a potential of mean force [54]. The expression of the potential energy does not rely a physical description but on a statistical one, and it has been admitted that the amino acid level is sufficient as far as the early stages of fold recognition or threading are concerned [55].The Metropolis criterion is used to accept or reject the new conformation. This simulation is stopped after 10^6^ to 10^7^ Monte Carlo steps, depending on the sequence length. This duration corresponds to the formation of some local clustering around hydrophobic residues and it is sufficient to capture the early steps of the intra chain interactions, which will presumably further on produce the folding nucleus at the transition state. To quantify these interactions, the number of non-covalent neighbours (NCN) is periodically recorded at each position. Two amino acids are assumed to be interacting (neighbours) if the distance between their alpha carbons is smaller than 5.9 Å. This limit of non-covalent interactions corresponds to the vector (2,2,2), i.e. the cut-off is slightly beyond the shell of second neighbours [30]. It is coherent with the values determined by Onofrio et al [56] as the cut-off between short and medium range interactions or with the characteristics of the hydrophobic core by Noel et al. [57]. The simulation is performed 100 times, starting from independent initial conformations. Then, the mean NCN is calculated at the end of the simulation. Positions where NCN is above 6 are called MIR (Most Interacting Residues) and positions where it is below 2 are called LIR (Least Interacting Residues), respectively. One example of the NCN distribution is given in Fig 2 for one protein of the Immunoglobulin-like fold, with CATH code 1svcP02. In some cases several consecutive MIR are obtained along the sequence, with no physical meaning if one admits an accuracy of the prediction of the order of ±1 amino acids. To avoid this effect, a smoothing procedure can be applied, giving rise to smoothed MIR. This option is available on the web server performing the MIR calculation (http://sprouts.rpbs.univ-paris-diderot.fr) [58]. Statistically, MIR are hydrophobic amino acids (in the list: FILMVWY), located in the bulk of the globular protein domains, and LIR are mainly located in the loops connecting regular secondary structures. We have previously shown that MIRs statistically match positions occupied by at least 75% of hydrophobic amino acids in structural alignments of highly divergent proteins (with sequence identities below 30% for any pair) [30]. These positions are actually related to the folding nucleus according to Poupon et al. [59–61].

### Hydrophobic core prediction by the “Fuzzy oil drop” model

The “fuzzy oil drop” (FOD) model is based on the comparison between distributions of theoretical and observed hydrophobicity densities in proteins [25]. Theory assumes an idealized hydrophobicity density distribution, with the highest value in the centre of the molecule, decreasing down to zero at the surface. This idealized (expected) hydrophobicity density, H^t^_i_, for the i-th amino acid is represented by a three-dimensional Gauss function:

$${\tilde{H}}_{i}^{t}=\frac{1}{{\tilde{H}}_{sum}^{t}}\exp\left(\frac{-{\left(x_{i}-\bar{x}\right)}^{2}}{2\sigma_{x}^{2}}\right)\exp\left(\frac{-{\left(y_{i}-\bar{y}\right)}^{2}}{2\sigma_{y}^{2}}\right)\exp\left(\frac{-{\left(z_{i}-\bar{z}\right)}^{2}}{2\sigma_{z}^{2}}\right)$$ (1)

The point $\left(\bar{x},\bar{y},\bar{z}\right)$ is the position of the geometric centre of the protein in the 3D coordinate system, placed in its origin, at (0,0,0). The protein should be rotated, making the line linking longest distance between two effective atoms in the molecule coaxial with (say) X-axis. It is then rotated around the X-axis to make the line linking the two most distant positions of the projections of effective atoms on the (say) YZ plane coaxial with Y-axis. Three parameters σ_x_, σ_y_, σ_z_ represent standard deviations of the size of the protein, equal to 1/3 of the highest absolute values of x-coordinate, y-coordinate and z-coordinate respectively (according to the 3-sigma rule), each increased by a constant of 9 Å [62]. The normalizing coefficient ${\tilde{H}}_{sum}^{t}$ represents the sum of all ${\tilde{H}}_{j}^{t}$ values of amino acids of the protein, making the ${\tilde{H}}_{i}^{t}$ value unit-less. The only input information for the theoretical distribution is a geometrical term concerning the full protein, i.e. the size of the ellipsoid “drop”, containing the protein, and characterized by σ_x_, σ_y_, σ_z._ Traditionally, value of the Gauss function (Eq 1) is interpreted as a hydrophobicity density (HD) probability. To be more precise, we can interpret it as the value of probability of high hydrophobicity density, but to make long name short, we will call ${\tilde{H}}_{i}^{t}$ a theoretical hydrophobicity density (HD).

The observed (empirical) hydrophobicity density (H^e^_i_) describes the effect of local inter-residual hydrophobic interactions of amino acid *i* with the protein. Values of this distribution can be calculated according to the Levitt function [62]:

$${\tilde{H}}_{i}^{e}=\frac{1}{{\tilde{H}}_{sum}^{e}}\underset{j}{\sum }\{\begin{array}{c} \left(H_{i}^{r}+H_{j}^{r}\right)\left(1-\frac{1}{2}\left(7{\left(\frac{r_{ij}}{c}\right)}^{2}-9{\left(\frac{r_{ij}}{c}\right)}^{4}+5{\left(\frac{r_{ij}}{c}\right)}^{6}-{\left(\frac{r_{ij}}{c}\right)}^{8}\right)\right)\text{for}\;r_{ij}\leq c\\ 0\;\text{for}\;r_{ij}>c \end{array}$$ (2)

where ${\tilde{H}}_{i}^{e}$ denotes the empirical interactions of the *i-*th residue with all other *j* residues satisfying the condition that inter atomic distances (*r*_*ij*_) are smaller than the *c* cut-off value, assigned at 9 Å according to Levitt. The idea is to consider all interactions within a radius of *c*, centred on the residue of interest, *i*. $H_{i}^{r}+\;H_{j}^{r}$ are hydrophobicity parameter values of the i-th and j-th residues, obtained from a hydrophobicity scale taken from [34]. ${\tilde{H}}_{sum}^{e}$ in Eq 2 is also a normalisation coefficient, giving rise to a value of ${\tilde{H}}_{i}^{e}$ with no unit, so one can directly compare both theoretical ${\tilde{H}}_{i}^{t}$ and observed ${\tilde{H}}_{i}^{e}$ HD distributions for each amino acid. The rationale behind the “fuzzy oil drop” (FOD) model relies in the best fit between observed and expected HDs, that will be developed below. It thus allows for recognition of residues constituting the hydrophobic core, the ones with high values of both expected and observed hydrophobicity density.

**Fig 3:**
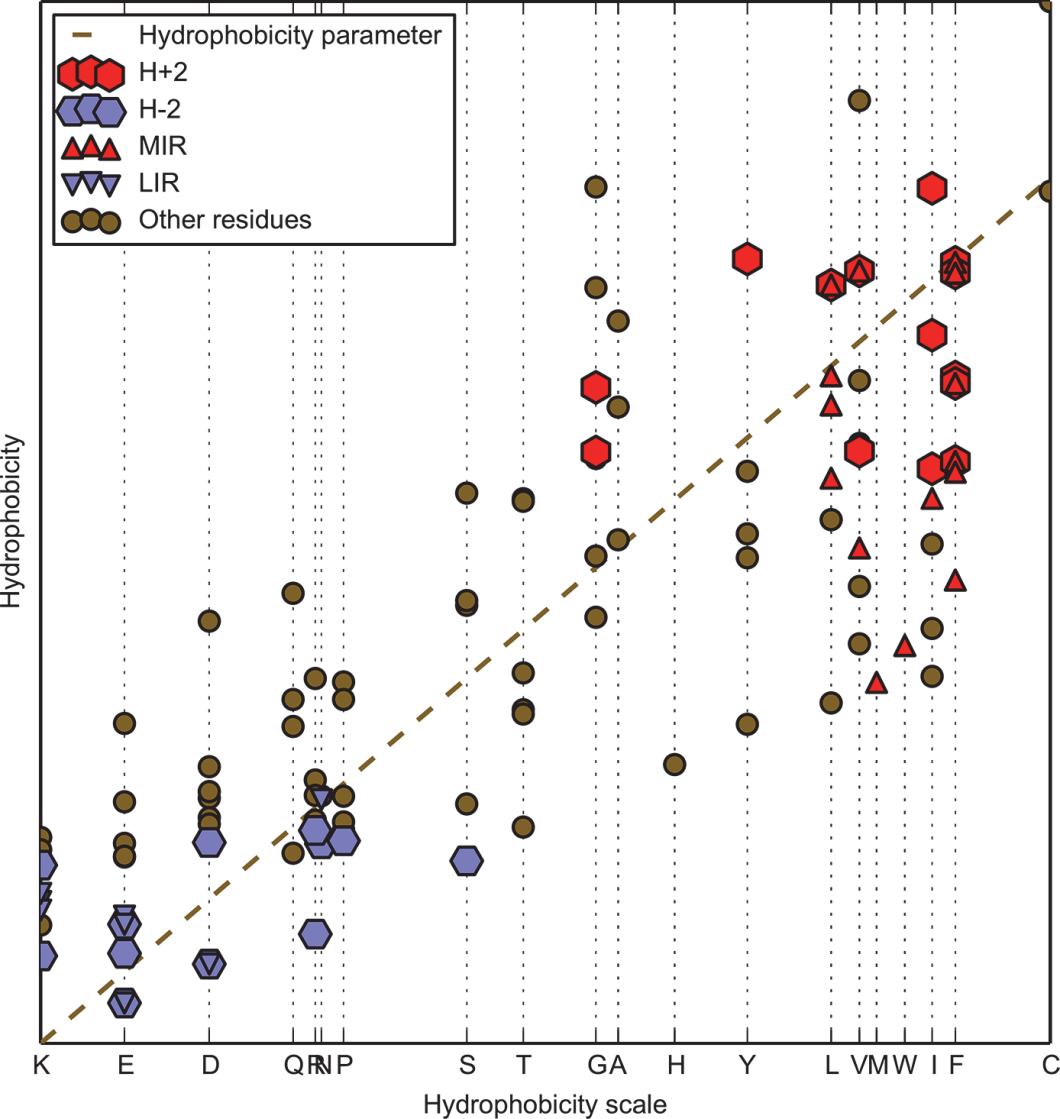
Immunoglobulin-like fold domain of CATH code 1svcP02, taken as an example to visualize to what extent the hydrophobicity of each amino acid ($H_{i}^{r}$) is influenced by the local hydrophobic interactions with surrounding residues (${\tilde{H}}_{i}^{e}$). Individual amino acids on the X-axis are ordered according to the value of hydrophobicity in the hydrophobicity scale [34]. This illustrates the difference between hydrophobicity linked to the amino acid on its own and the one once it is included in the structure of a protein. All residues, including those classified as MIR, LIR, highly hydrophobic (H+2) or highly hydrophilic (H–2) are presented in forms of different markers. Dashed diagonal line defines observed hydrophobicity values equal to the hydrophobicity parameters from the scale.

One must carefully distinguish between hydrophobicity of an amino acid given in a scale (H^r^_i_) and hydrophobicity density, calculated for theoretical (Eq 1) or observed (Eq 2) distributions. The hydrophobicity value attached to each amino acid is a constant value, which depends only on the scale chosen for analysis, and is independent of the environment. The theoretical hydrophobicity density (${\tilde{H}}_{i}^{t}$) is the expected idealized form of hydrophobic core calculated using the function given in Eq 1, and is independent of any hydrophobicity scale. From hereon, we will call it theoretical HD. On the contrary, the observed hydrophobicity density (${\tilde{H}}_{i}^{e}$), as calculated using Eq 2, represents the collection of hydrophobic characteristics of a particular amino acid within the local environment of residues limited at a distance of 9 Å (cut-off distance for hydrophobic interaction) around the central residue. From hereon, we will call it observed HD. Fig 3 shows the distribution of $H_{i}^{r}$ and ${\tilde{H}}_{i}^{e}$ on the example of a 105-amino acid protein domain of immunoglobulin-like fold (CATH code 1svcP02). This is aimed at illustrating the effect induced by the environment on a scale that is strictly constant and unaffected by the interactions due to a particular conformation. It shows that the observed HD is not constant and can either increase or decrease its value depending on the local environment of the considered amino acid. This can be seen from the departure of the points of Fig 3 relative to the dashed line.

The classification of the HD status of a residue according to FOD model is based on the agreement between theoretical and observed HD values of this particular amino acid. High values of both theoretical and observed hydrophobicity densities of a residue classify it as belonging to the hydrophobic core. Low values of both expected and observed hydrophobicity density suggest that the residue is localized on the surface and in contact with solvent or with other amino acids of low hydrophobicity. Therefore, the key point is the coherence between the observed and theoretical densities for a given residue, either both high or both low.

**Fig 4:**
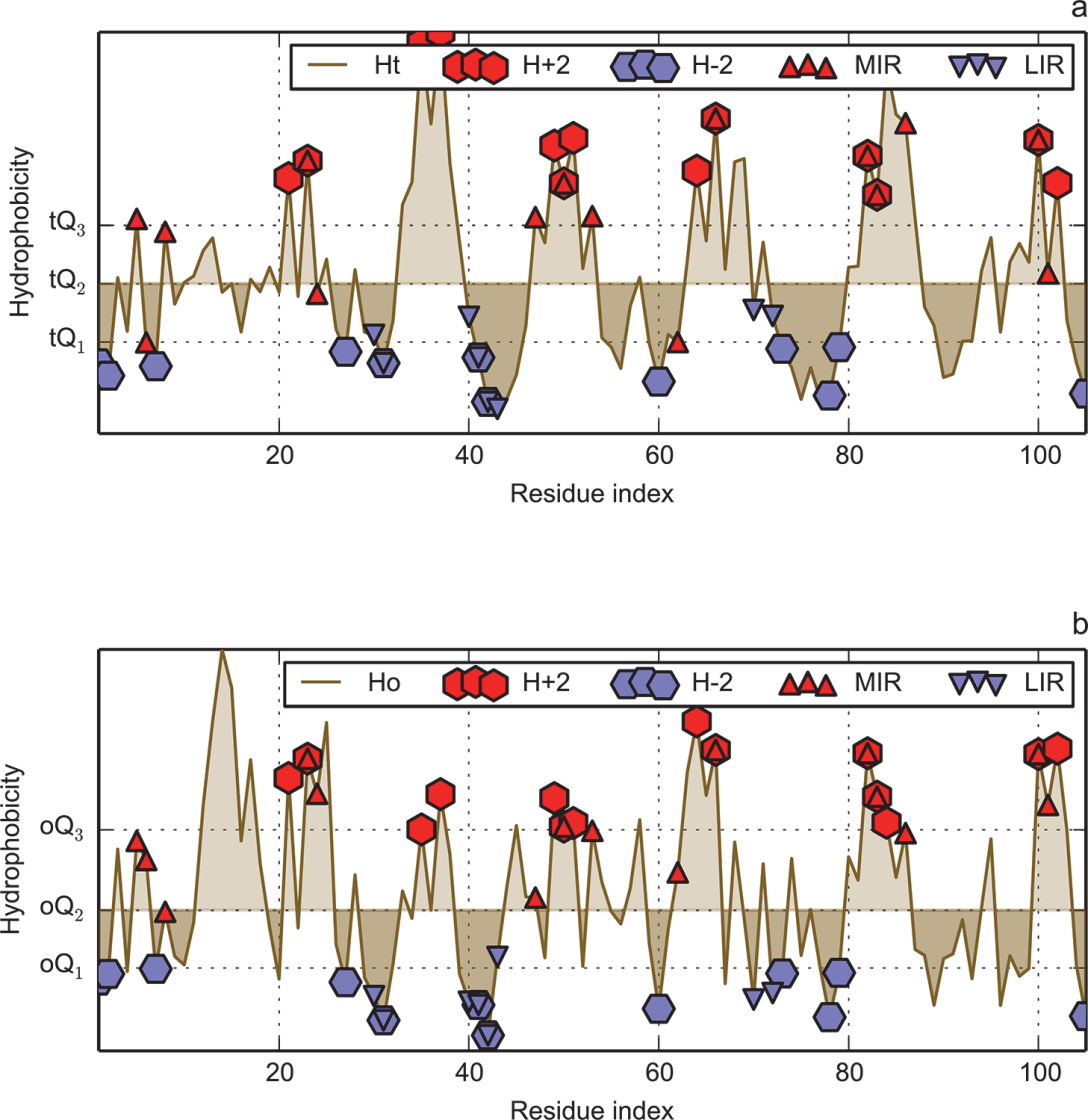
Hydrophobicity density distributions for immunoglobulin-like fold of CATH code 1svcP02, taken as an example. X-axis represents the sequence of this protein. Y-axis represents hydrophobicity density: theoretical (a) and observed (b). The Q symbols visualise positions of appropriate quantiles, with prefix “t” for theoretical and “o” for observed distributions. These thresholds are used to classify residues into four FOD groups: hydrophobic (H+2, H+1) and hydrophilic (H–2, H–1). Some residues may be left unassigned. For information on calculation of these thresholds, see Table 2. Residues classified as MIR, LIR, highly hydrophobic (H+2) or highly hydrophilic (H–2) are presented with different markers.

Hydrophobicity density profiles in FOD model depend on the protein chain under consideration. Instead of a fixed cut-off, which fails to be universal, quartiles of the distributions of HD along the sequence are taken as thresholds for classification of a particular residue. Three quartiles are taken into consideration: Q_1_ which discriminates the residues of lowest 25% of hydrophobicity density values; Q_2_ which corresponds to 50% level of hydrophobicity density (median) and Q_3_ which discriminates the highest 25% of hydrophobicity density values. Each distribution has its own set of these three quartiles, prefixed with either “t” for theory or “o” for observation, as visualized on Fig 4, also for the immunoglobulin-like domain 1svcP02. Details of the classification criteria, which are shown in Table 2, define four hydrophobicity classes: two hydrophobic (“high” / H+2, and “all” / H+1) and two hydrophilic (“high” / H–2, and “all” / H–1); “all” classes contain the “high” classes. Nomenclature presented in Table 2 is used throughout this paper. Suppose for instance that both theoretical and observed hydrophobicity values fall above Q_3_ of their respected distributions. Then, this particular amino acid will be warded a YES for the H+2 and H+1 FOD classes in the contingency tables that will be further presented. If observation is above Q_2_ and theory above Q_3_ (or vice-versa) then such residue in the contingency tables would receive a NO for the FOD class H+2 but still a YES for H+1 class. Therefore, for each residue, a qualitative criterion is established indicating if (and how) it follows or not the FOD model.

**Table 2:** Naming conventions and hydrophobicity density requirements for FOD model classes.

FOD hydrophobicity class			Hydrophobicity density	
Name	Description		Theoretical	Observed
H+2	high	hydrophobic	Ht > tQ_3_	Ho > oQ_3_
H+1	all	hydrophobic	Ht > tQ_2_	Ho > oQ_2_
H–1	all	hydrophilic	Ht < tQ_2_	Ho < oQ_2_
H–2	high	hydrophilic	Ht < tQ_1_	Ho < oQ_1_

Example levels of Q quantiles are visualized in Fig 4. One reads as follows: if hydrophobicity of an amino acid is above Q_3_ for theory and observation, then it is assumed to be highly hydrophobic and assigned to both H+2 and H+1 classes. If values of both theory and observation are below second quantile Q_2_ of their respected distributions, such residue is marked as hydrophilic and assigned to H–1 class. Residues with hydrophobicity characteristics not matching any of the criteria, such as those with Ht > tQ_2_ and Ho < oQ_2_ (or vice versa), are assumed to be discordant with the FOD model and are left unassigned.

### Combination of methods

The degree of IR (MIR or LIR) status enrichment for a given FOD class (H+2, H+1, H-2, H-1) and reciprocally, relies on the non-random behaviour of their intersection. It can be quantitatively assessed by the hypergeometric distribution [63]. This discrete probability distribution describes the probability of *k* successes in a sample constituted by *n* draws without replacement, from a finite population of size *N* containing exactly *K* successes. The density of this distribution, with parameters *K*, *N-K* and *n*, is given by:

$$P\left(X=k\right)=\frac{\left(\begin{array}{c} K\\ k \end{array}\right)\left(\begin{array}{c} N-K\\ n-k \end{array}\right)}{\left(\begin{array}{c} N\\ n \end{array}\right)}$$ (3)

for *k* = 0, …, *n*

In this paper, *N* represents the total number of amino acids of the given protein dataset (Ig-like or Flav fold), *n* is the total size of the given FOD class (i.e. the total number of amino acids which are considered to be drawn without replacement from the population), *K* is the total number of MIR or LIR in the given amino acid population, of which *k* are part of the sample (i.e. belong to the given FOD class). In other words, *k* represents the size of the IR/FOD intersection.

The hypergeometric distribution gives the probability that the given FOD class shares exactly *k* residues with the given IR status by chance, and reciprocally. The probability (p-value) of observing an overlap of given IR status and FOD class of size greater than or equal to *k* by chance is calculated according to the hypergeometric cumulative density function, as:

$$P\left(X\geq k\right)=1-\sum_{x=0}^{k-1}P\left(X=x\right)$$ (4)

This probability decreases (from 1 to 0) while *k* increases. If this probability is sufficiently low (the threshold is classically set to 0.05), then the given FOD class is considered enriched for the given IR status (and reciprocally). In such a case, the statistical significance of the IR/FOD linkage is confirmed, the size of their intersection differing from random.

We compared the results of MIR and FOD methods for each analysed protein domain, for MIR and H+2, MIR and H+1, LIR and H–2, LIR and H–1. The reason to perform the intersection between results of the two approaches is to reduce the number of false positive members of the fold coding residues. These selected residues are compared to the experimental data, available for four immunoglobulin-like fold studied by the group of Jane Clarke [19,39,64,65] and for two flavodoxin folds [44,45]. The sensitivity (or true positive rate) and specificity (true negative rate) have been calculated for each method (IR and FOD) separately and for their intersection, in order to evaluate their ability to identify the experimentally determined key residues. For a given method (or their intersection), sensitivity and specificity quantify the proportion of the nucleus residues and the proportion of the non-nucleus residues that are correctly predicted as such, respectively. These rates are computed as follows:

True Positive Rate(Sensitivity),TPR=TP/(TP+FN) (5)

True Negative Rate(Specificity),TNR=TN/(TN+FP) (6)

TP, FP, FN and TN are respectively: true positive, false positive, false negative and true negative counts.

Since we aim at identifying the key residues that can be considered as the signature of a fold, and giving rise to the folding nucleus, we computed the position-related proportion of residues predicted as MIR, LIR and/or FOD in the multiple sequence alignment of each domain dataset.

## Results

**Fig 5:**
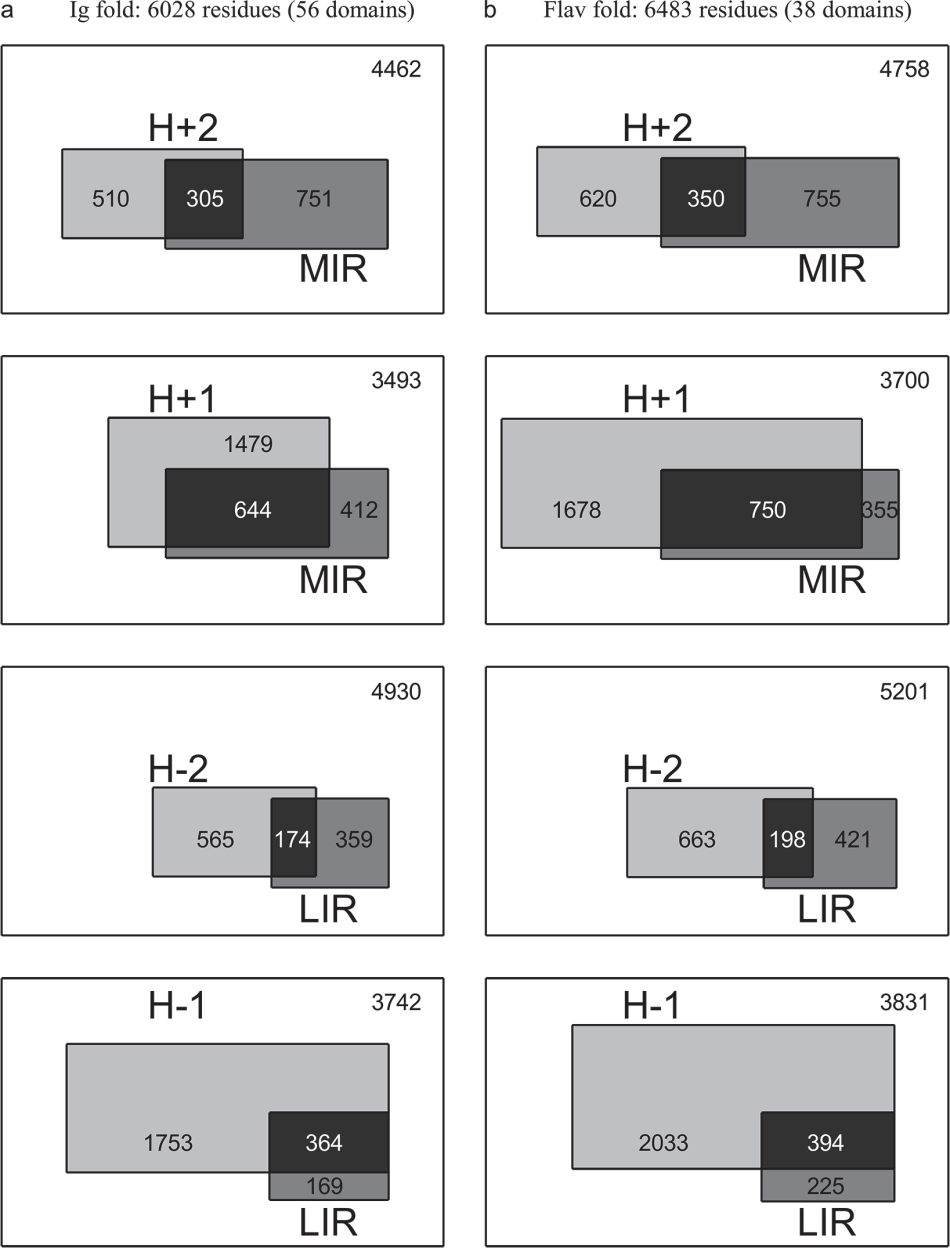
Schematic representation of the fold-level relation between FOD classes (hydrophilic: H-2, H-1; hydrophobic: H+2, H+1) and MIR/LIR status of residues from domains belonging to the immunoglobulin-like (A) and flavodoxin (B) folds. The number of residues of each category is represented by a scaled rectangle area. For example, one should read the top left chart as follows: the number of H+2 and not MIR is 510; the number of MIR and not H+2 is 751; the number of both MIR and H+2 is 305; the number of neither MIR nor H+2 is 4462.

**Fig 6:**
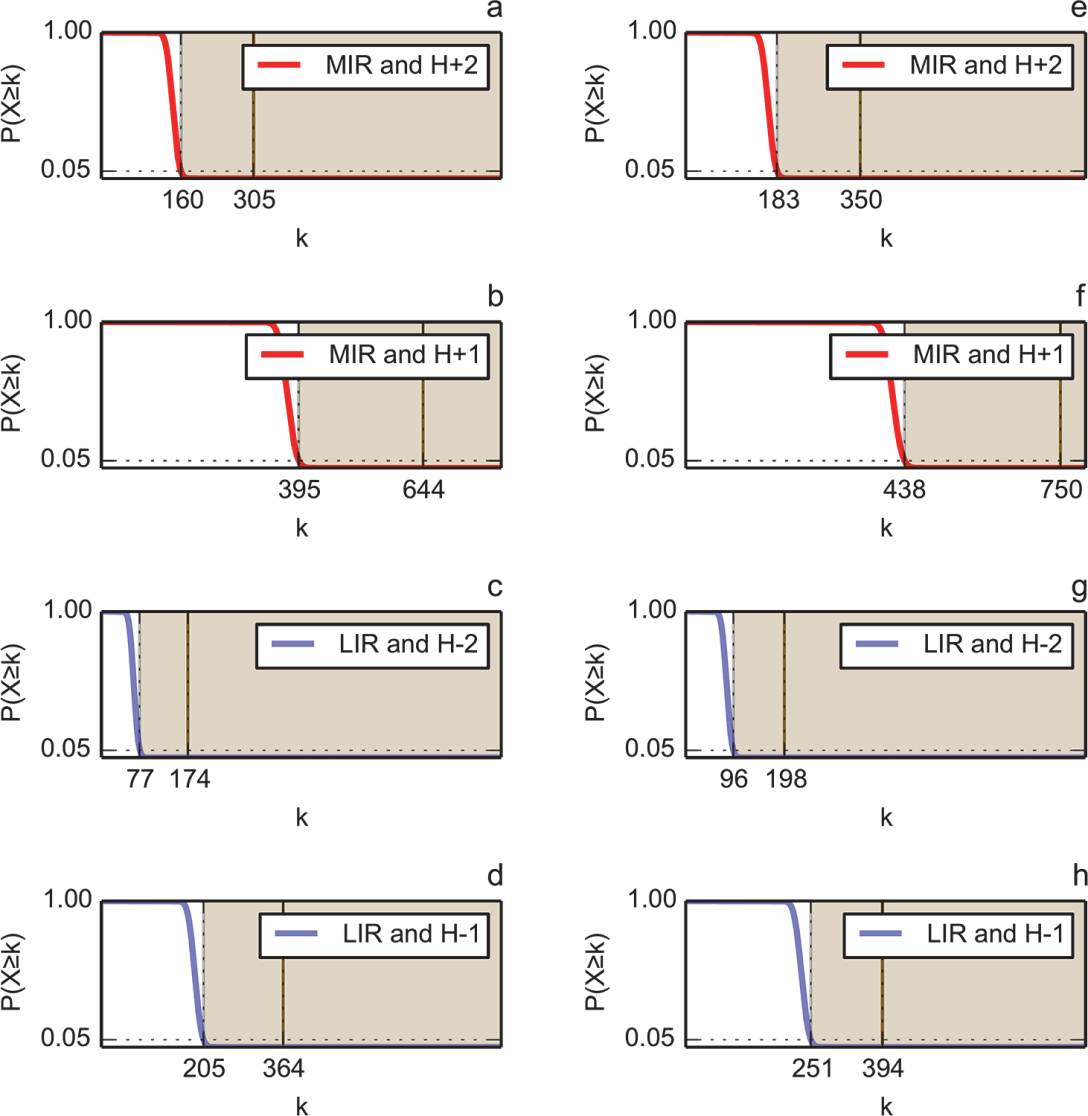
Probabilities of observing an IR/FOD intersection of size greater than or equal to *k*, derived from the parametric hypergeometric distributions, for the Ig (a-d) and Flav (e-h) folds. The values of the parameters (*K*, *N-K* and *n*) of the hypergeometric distributions are given in Table 3. The probability decreases from 1 down to 0 while *k* increases. The dashed vertical line represents the 95% quantile, i.e. the theoretical value of *k* that corresponds to a p-value exactly equal to 0.05. It constitutes the lower limit of the significance area (grey shading), which covers the values of *k* that correspond to an IR/FOD intersection significantly different from random. The continuous vertical line represents the observed size of the IR/FOD intersection, i.e. the observed value of *k*. These values (95% quantile, observed intersection size) are given in Table 3.

Fig 5 compares on one hand the number of MIR with the number of hydrophobic FOD (H+2 or H+1 classes), and on the other hand the number of LIR with the number of hydrophilic FOD (H-2 or H-1 classes) for the set of domains from the immunoglobulin-like (Ig) and flavodoxin (Flav) folds. The size of each rectangle area is proportional to the corresponding number of residues. This picture is proposed to visualise the proportion of the intersecting regions (in black), compared to the FOD and MIR classes on their own. So it gives an overview of the interest of combining the two methods to reduce the number of residues susceptible to produce a signature of the fold. In order to quantitatively measure the degree of accordance between FOD and MIR methods, we used hypergeometric distributions to evaluate the significance of their intersection. A p-value has been computed for each IR/FOD relevant combination of each fold dataset. It gives the probability that an IR/FOD intersection of size greater than or equal to the observed one would occur by chance. The distribution parameters and the corresponding p-values are given in Table 3 (fold-level analysis) and S1 Table (domain-level analysis), and the theoretical p-values as a function of the IR/FOD intersection size parameter are plotted in Fig 6. The fact that the computed size of the intersection is far above the random threshold (Fig 6) assesses the ability of joint MIR and FOD methods to decipher a reduced set of residues typical of the core and/or the nucleus of a fold. Let us also notice that residues both predicted as least interacting (LIR) and assigned as hydrophilic (H-1 or H-2) constitute events with a probability of occurrence significantly higher than a random drawn.

**Table 3:** Parameters of the hypergeometric distributions used to assess the statistical significance of the IR/FOD intersections for immunoglobulin-like (Ig) fold (A) and flavodoxin (Flav) fold (B).

**A**						
	N	n	K	k	p-value	95% quantile
H+2/MIR	6028	815	1056	305	< 2.22e-16	160
H+1/MIR	6028	2123	1056	644	< 2.22e-16	395
H-2/LIR	6028	739	533	174	< 2.22e-16	77
H-1/LIR	6028	2117	533	364	< 2.22e-16	205
**B**						
H+2/MIR	6483	970	1105	350	< 2.22e-16	183
H+1/MIR	6483	2428	1105	750	< 2.22e-16	438
H-2/LIR	6483	861	619	198	< 2.22e-16	96
H-1/LIR	6483	2427	619	394	< 2.22e-16	251

*N* represents the total number of amino acids from domains belonging to the given fold (population size), *n* is the total size of the given FOD class (sample size), *K* is the total number of MIR or LIR in the amino acid population (number of successes in the population), of which *k* belong to the given FOD class (number of successes in the sample, or size of the IR/FOD intersection). For a given set of parameters, the hypergeometric distribution is used to compute a p-value, i.e. the probability of observing an IR/FOD intersection of size greater than or equal to *k* by chance. The p-value significance threshold is set to 0.05 (for theoretical evolution of the p-value as a function of *k*, see Fig 6); the 95% quantile represents the size of the IR/FOD intersection which would correspond to a p-value exactly equal to 0.05, according to the given hypergeometric distribution.

We compared the residues selected by the two methods (IR and FOD) to those experimentally identified as part of the folding nucleus in four proteins with Ig fold on one hand and attributed to the core of two proteins of Flav fold on the other hand (see below for details on these protein domains and experimental results). The sensitivity and specificity of both methods are presented in Table 4. The combination of MIR and FOD methods result in a simultaneous decrease in sensitivity and increase in specificity. For each of these domains, the specificity of the H+2/MIR method reaches at least 0.95, indicating that at most 5% of the non nucleus residues are predicted as part of the nucleus or core by the intersection of our methods.

**Table 4:** Sensibility (Sens / True Positive Rate) and specificity (Spec / True Negative Rate) of both methods (FOD and MIR) separately and jointly, according to the experimentally-determined folding nucleus or core as reference for four domains of the Ig fold and two domains of the Flav fold.

		MIR		H+2		H+1		H+2/MIR		H+1/MIR	
Fold	Domain	Sens	Spec	Sens	Spec	Sens	Spec	Sens	Spec	Sens	Spec
Ig	1k85A00	0,750	0,845	**1,000**	0,845	**1,000**	0,643	0,750	**0,964**	0,750	0,881
	1tenA00	0,500	0,860	0,250	0,895	**1,000**	0,721	0,000	**0,965**	0,500	0,953
	1titA00	0,250	0,882	**1,000**	0,871	**1,000**	0,659	0,250	**0,953**	0,250	0,941
	1ttfA00	0,625	0,884	0,750	0,895	**1,000**	0,709	0,375	**0,977**	0,625	0,919
Flav	1eayA00	0,556	**0,967**	0,389	**0,967**	0,778	0,848	0,250	**0,989**	0,444	**0,967**
	1ftgA00	0,381	0,844	0,429	0,844	0,571	0,633	0,381	**0,946**	0,381	**0,871**

For each domain, the two highest values are highlighted.

**Fig 7:**
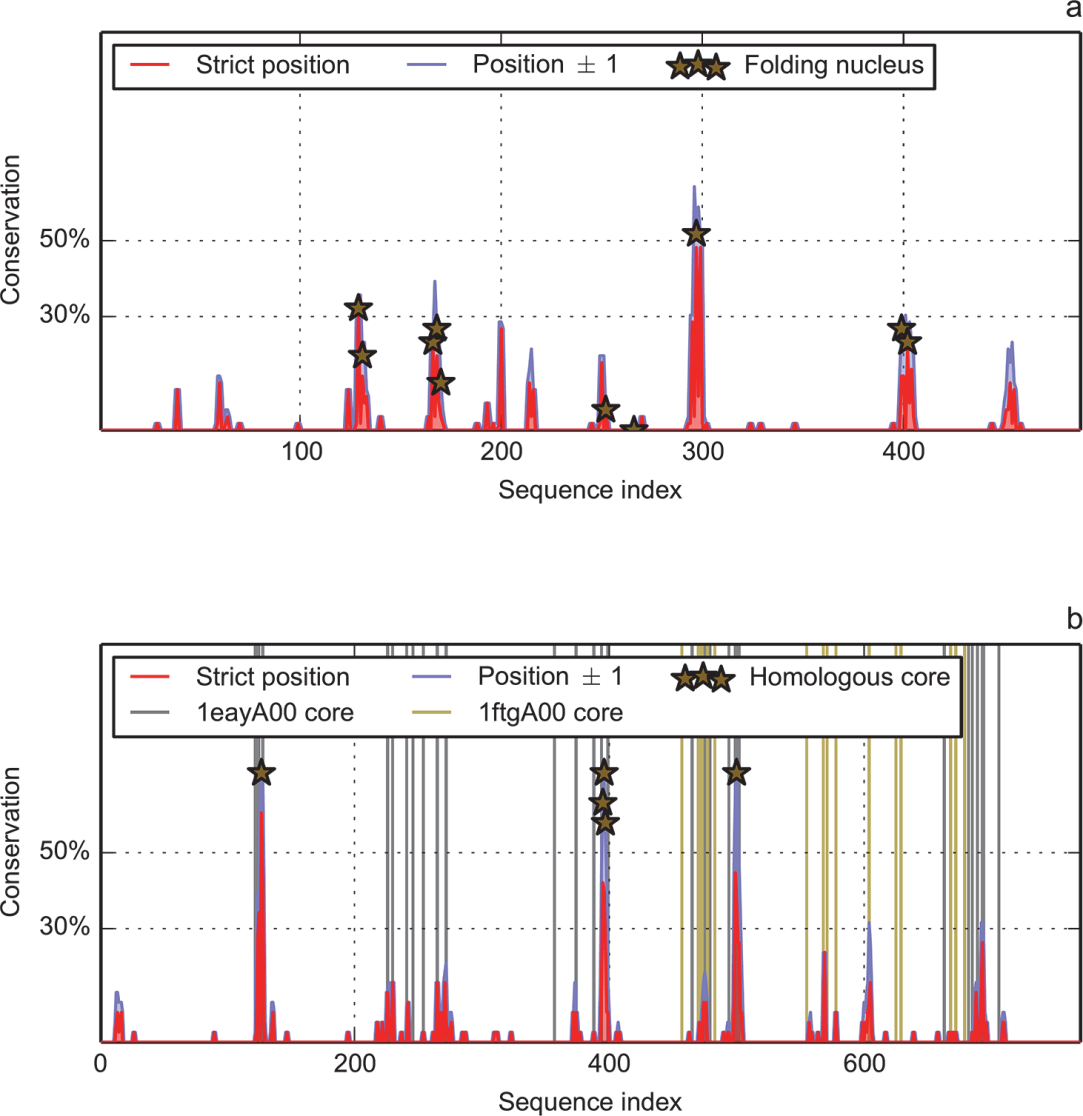
Position-related conservation of MIR and FOD (H+2) predictions in the multiple sequence alignment. The X-axis represents the sequence positions in the multiple alignment, therefore it is much longer than a mean domain size to account for the numerous insertions/deletions. At a given position in the alignment, the conservation of predicted MIR and H+2 is expressed as a proportion of total number of sequences. Two windows are used: strict position (length 1) in red and position ±1 (length 3) in blue. a) Ig fold. Markers indicate positions experimentally determined as members of the folding nucleus in four CATH domains: 1tenA00, 1titA00, 1ttfA00 and 1k85A000. b) Flav fold. Vertical green (brown) lines represent the positions attributed to core of 1eayA00 (1ftgA00). Markers indicate positions in the alignment attributed to the core of both 1eayA00 and 1ftgA00.

In order to better evidence the effect of the combination of MIR and FOD methods on the number of key residues one can consider as a signature of a fold, we present in Fig 7 the percentage of residues both MIR and H+2 in the multiple sequence alignment of the two datasets. We estimate the prediction of the MIR positions to be ±1. Therefore, a given position is attributed as MIR and H+2 if either one of its two neighbours or itself (±1) is predicted to belong to the intersection of results of the two methods. This produces results, shown in Fig 7A for the Ig fold, with a very small number of conserved positions (peaks) in the distribution. If we admit to put a threshold at 30% (i.e. 30% of the 56 sequences of the alignment), four peaks are present in the prediction of the nucleus of the Ig fold by the combination of the two methods. The threshold at 30% can obviously be questioned, and to get rid of this arbitrary decision, the same study should be performed on a large number of folds to obtain a value lying on statistical outputs, out of reach of the present proof of concept. Nevertheless, this is promising because it is coherent with several experimental evidences that show the conservation of the transition state ensemble (TSE) among members of the Ig fold [46]. This conclusion matches the fact that the number of conserved MIR/H+2 positions is small.

One is now willing to compare this plot to the positions experimentally admitted as belonging to the nucleus. Four domains with immunoglobulin-like fold have been widely studied by the group of Jane Clarke [19,39,64,65]. Although Phi values [66] do not fully determine the nucleus, they nevertheless present a weak correlation with the number of inter residue contacts [27]. So an analysis of the broken interactions has been accounted for by the Jane Clarke group. Positions of the folding nucleus are represented in Fig 7A by markers (stars). Correspondence between the multiple alignment position and the PDB (Protein Data Bank) numbering of these residues is given in S2 Table. For a fibronectin type III domain (CATH code 1tenA00), four residues over 90 constitute the putative folding nucleus (in the following, residue numbers refer to their PDB numbering): I821, Y837, I860 and V871 [39]. Our recognition of this nucleus is L835, L873 and I874. If we accept a precision of the prediction of ±1 residues, we are partially consistent with these experimental results. Let us notice that I860 and V871 are MIRs and are both recognized as members of hydrophobic class H+1. Concerning I821, which is not recognized as MIR, it is nevertheless in the H+2 class. In a second fibronectin of type III, (CATH code 1k85A00) [19], the folding nucleus is composed of L578, V594, I611 and V624. The three first ones are MIR and all are H+2, so only one is missing. The last fibronectic type III studied, (CATH code 1ttfA00), has been attributed the following folding nucleus: Y32, I34, Y36, F48, V50, I59, I70 and V72 [64]. The majority of them are MIR at the exception of Y36, V50 and I59, which are only H+2. For the 27th Ig repeat of the human cardiatic titin (CATH code 1titA00) [65], the folding nucleus is composed of I23, W34, L58 and F73. In this last case, agreement with our prediction is rather low, since while all of them are classified by FOD model as highly hydrophobic, only L58 is both a MIR and H+2. Among these four domains with an experimentally determined domain, one must notice that all of them have a nucleus residue at position 297 of the alignment (S2 Table). If we take the amino acids predicted by the combined method as a reference, all the peaks present in more than 30% of the sequences (Fig 7A), at positions 130±1, 169±1, 297, 399 and 402, do correspond to nucleus residues. Positions 166, 252 and 266, predicted as not belonging to the folding nucleus, are actually in the experimental one. One must mention that they are all specific of the folding nucleus of the domain 1ttfA00, which has the peculiarity of having a nucleus that contains a double number of amino acids, compared to the three others. So the set of residues coding for the fold, resulting both from the combination of the methods, and from the use of a multiple alignment, has been decreased to five positions.

If one now looks at the combined methods (residues both predicted as a MIR and assigned to the H+2 class) for the flavodoxin dataset represented in Fig 7B, there are three peaks at levels of conservation higher than 30%. Experimental Phi values have been published for two members of the Flav fold: CheY (CATH code 1eayA00), a 128 aa (amino acid) domain with a kinetic intermediate along the folding pathway [45] and the *Anabaena* apoflavodoxin (CATH code 1ftgA00), a 168 aa domain [44]. For 1eayA00, in neutral conditions, the folding is a two state process, but under denaturant conditions it follows a two states model. All Phi values are rather small for the wild type, and only two positions are larger than 0.5, amazingly both occupied by an aspartate (D12 and D57). One point mutation F14N allows to approximate the folding by a two state behaviour [45]. Then mutations performed on this pseudo wild type protein provide larger values of Phi, and in particular greater than 0.5 for seven positions: V10, V11, V33, A36, D38 (if it is mutated to glycine), A42, and V54. This peculiar structure of the transition state has been analysed as the first half partly folded, without any clear cut between core and non core residues, and the second part completely disorganised. The apoflavodoxin 1ftgA00 is also characterized by strange Phi values, that may be higher than 1 for non-hydrophobic residues (S71, E107). These remarks indicate a diffuse nature of the nucleus [44] and a set of intermediate states with a low compacity [42]. Therefore, we preferentially use the word core instead of nucleus to qualify this fold, following the literature. At his point, one might expect a lower conservation of the positions of the key residues of the Flav fold, compared to the Ig fold. If one considers the positions where 1eayA00 and 1ftgA00 share an intersection of MIR/H+2, in five cases they are located above the 30% threshold (Fig 7B): roughly at alignment positions 125, 400, 500, 600 and 650. This shows that this method captures some of the diffuse aspect of the nucleus in the flavodoxin fold: the three highest peaks (at more than 60% conservation) in the intersection of MIR and FOD methods actually correspond to positions where experimental results indicate a core residue for both 1eayA00 and 1ftgA00 (in red in S2 Table). The two small peaks at 30% conservation correspond to experimental core position from one of these two proteins. Therefore, one may admit these peaks as a signature of the Flav fold, although it may be partial. Besides, one must remind that the Flav fold has two cores, and the level of conservation may not necessarily be the same for each of them.

**Fig 8:**
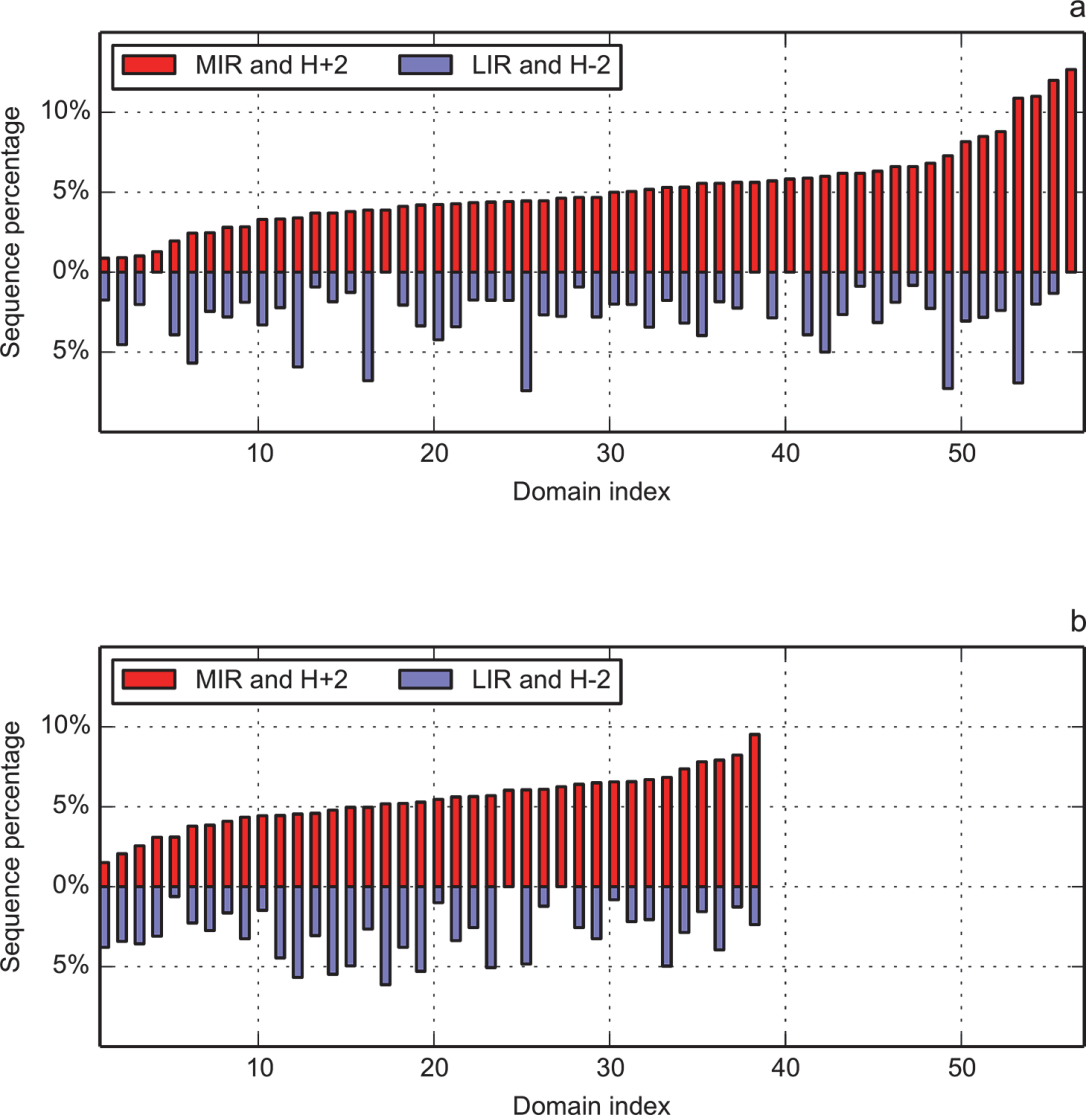
Relation of the MIR/H+2 (red bars) and LIR/H–2 (blue bars) combinations with the sequence length for domains from immunoglobulin-like (a) and flavodoxin (b) folds. In each fold, average length ratio is 5% for MIR and H+2, and 3% for LIR and H–2. Domains on the X-axis are sorted in ascending order by their MIR percentage.

Fig 8 presents the percentage of sequence lengths classified as both MIR and highly hydrophobic (H+2) and as both LIR and highly hydrophilic (H–2) for the two folds. The abscissa is an index number that sorts the individual proteins by increasing values of their MIR/H+2 percentages. Mean values are the following: 5.1% (standard deviation, sd, 2.5%) MIR in Ig; 5.4% (sd 1.7%) for MIR in Flav; 2.7% (sd 1.8%) for LIR in Ig and 3.0% (sd 1.6%) for LIR in Flav. There is clearly no correlation between MIR/H+2 and LIR/H–2 distributions, indicating their independent behaviours. Actually, the presented methods are more focused on the prediction of the core or nucleus rather than on the identification of non-interacting residues, that can be located in parts of the proteins not necessarily linked to hydrophobicity. On the contrary, this is very seldom that a buried residue is not hydrophobic. Therefore, the predictive power of MIRs is more informative than for LIRs. Besides, value range of MIR/H+2 proportion is rather large in the immunoglobulin-like fold (Fig 8A), going from 1% up to 12%, which explains its higher standard deviation.

## Discussion

We have predicted the positions of a set of intra-domain interacting residues, the MIRs, expected to some extend to contain the positions that will form the so-called nucleus in the transition state of the folding process. This is deduced from the mean number of non-covalent neighbours during a simulation where the chain is displaced to empty nodes of a cubic lattice. This study has been performed on two domain folds, each one containing a large number of very divergent sequences. For the immunoglobulin-like fold, 56 domains of known structure have a mean length 108 amino acids, and a mean number of 19 MIRs per domain [31]. For the flavodoxin fold, 38 domains of mean length 171 give birth to a mean number of 29 MIRs per domain. So, the proportion of MIR for these two folds is in the range [16–18]%, a ratio of the order of previous estimations of the folding nucleus published in the literature, although this later varies from a few per cents up to half the sequence. Experimental determination of the folding nucleus is not easy, since it needs to mutate each position assumed to be part of it. Therefore it requires long biotechnological investigations. Instead of looking to determine the “exact” size of the nucleus, considering that validation is tedious, we actually focused mainly on the minimum number of positions that are occupied by hydrophobic amino acids involved in numerous interactions. One can then consider them as a fold signature. One must also remind that the over estimation of the folding nucleus by the MIR method, if any, can rely on physical reasons. It has been established in certain cases that non-native interactions are necessary during the folding process and they may disappear in the late steps [19]. As it has been stated by Treptow et al., the folding nucleus “could be compared to a ladder that, after being used, can be thrown away” [67]. This has been later experimentally verified by NMR determination of a folding intermediate of a redesigned apocytochrome b562 [68]. Nevertheless, the debate is still open, and for instance it has been argued conversely that non native interactions do not play a significant role for almost all the proteins [69]. The simulation of the folding process on a lattice, especially in our case because we stop it at the early steps, may capture some of these intermediate contacts, which can vanish in the native structure. Increasing by several orders of magnitude the duration of the simulation, to catch long-range interactions, is out of reach for the moment, due to CPU constraints. Besides, we paid more attention on the analysis of folds than to the accurate prediction on a given chain. There is a second argument one can advance to explain the over prediction of interacting hydrophobic residues. Following the work by the group of Reynolds [70], one can find, at the surface of the domains, residues engaged in numerous interactions; these residues are located at patches where ligands bind to the proteins. Working with a set of structures of a common fold but various functions, gathers proteins with interacting surfaces that are presumably not conserved, while the deep core will be, by structural necessity.

In this paper, we aimed at decreasing the number of residues predicted to be a signature of the fold. This is why we proposed to take the intersection between the set of MIRs and the hydrophobic core, as identified by the “fuzzy oil drop” model. The FOD model assumes that a globular protein can be simulated by a 3D Gaussian distribution of hydrophobicity. Positions where both ideal (following the 3D Gaussian distribution) and observed hydrophobic density values are significantly high (according to the HD profiles) are classified as highly hydrophobic (belonging to H+2 class of the FOD model). Intersection of MIR and H+2 positions, which is statistically significant, improves the prediction by decreasing their numbers to the level of [3–5]% in each fold, (with standard deviation of 2.5% and 1.7%, respectively), instead of previous [16–18]%. On the same data set, the mean number of LIRs following the FOD model (residues classified as highly hydrophilic, H–2) is at the level of 3%. %. This, together with standard deviation being close to 1.7% in each fold, shows that LIR/H-2 distribution is more compact than that of MIR/H+2, however no correlation is observed between them.

The intersection of the two proposed methods allows evidencing a few positions conserved among the proteins once their sequences have been aligned. For both folds, the four or five most conserved positions are experimentally attributed to the core or nucleus. It means that the number of residues one can consider as a signature of the fold is very limited, in agreement with the experiments. So, the intersection of our methods significantly increases the specificity of the prediction. To test the limits of the method, let us return to the Paracelsus challenge. The group of John Orban [71–73] successfully produced pairs of domains with increasing levels of sequence identity while maintaining separate folds: a three helix bundle on one hand (called GA, CATH code 2lhcA00), and a four stranded sheet facing a helix on the other hand (called GB, CATH code 2lhdA00). They even conserved these two folds from a pair of sequences differing by a single amino acid (Leucine toward Tyrosine) over 56, therefore with a level of sequence identity of 98%. This is obviously very difficult to explain from a computational point of view since it reaches the limits of any predictive method. It has been deeply analysed by the group of van Gunsteren who concluded that structure and stability result from a complex combination of interdependent factors [74]. Our rule of thumb is that a length of 56 is a limit in size for MIR prediction, nevertheless a fine fluctuation of the hydrophobic density appears in the simulation, since one goes from 4 MIR in GA to 8 MIR in GB. One may assume that we are coherent with the argument of [73], which states that GA is less stable than GB. In order to get rid of statistical fluctuations, one compares the NCN smoothed distribution of these two domains (available at http://sprouts.rpbs.univ-paris-diderot.fr/mir.html). Peak in the NCN distribution of PDB code 2LHC at L45 for GA disappears when mutated to Tyrosine in GB while the global shape of the distribution is maintained [75,76]. Combination of MIR and FOD methods reinforces the hypothesis of stability: in GA, two positions are both registered as MIR and H+2, L45 and I49, while in GB, there are three, at L7, F30 and F52.

One may also ask the question of the effect of symmetry on the prediction of the key residues. Fold analysis has revealed a high prevalence of proteins presenting a structural symmetry [77,78]. Why does it happen so commonly? Following Longo et al., it may be due to repeated instances of key folding nucleus. One way to demonstrate this may be to perform circular permutations that can reveal, in case of failure of the folding, the presence of a region comprising a key folding nucleus. This has been done by the Blaber group for symfoil-4T [77], a 126 residue protein, assumed to be a 42-mer repeat through circular permutations. The native domain (CATH code 3o4bA00) is permuted at each of the three beta turns at its N terminal end (PDB or CATH codes are 3SNV, 3p6iA00, 3p6jA00 respectively). The resulting mutants show no effect on the kinetics of folding. In the analysis, the key subset of residues essential for folding is in the range [50–66]% of the overall fold. We find that the number of MIR in the four cases ranges from 26 to 30, close to one fourth of the sequence, and the number of MIR common to any of the permuted protein and the native one is rather stable, between 22 and 24, so most of them are conserved. Besides, when looking at the smoothed distribution of NCN (available on the MIR server at RPBS), peaks are just shifted to take into account the permuted fragment, but there is no other effect on the intra chain contacts. Actually, peaks in the smoothed distribution of NCN also correspond to positions marked as both MIR and H+2. This example indicates that our combined method of prediction of intra chain contacts is not sensible to long range interactions, and therefore the presence of symmetries in the structure is a second order effect, if any.

The group of Oliveberg also studied the circular permutations of the ribosomal protein S6 (CATH code 2kjvA00), belonging to ferredoxin-like fold, with four strands facing two helices [79,80,81,82]. In a succession of papers, it is shown that S6 is composed of two spatially adjacent nuclei, sharing the first strand. When looking at the distribution of the NCN for the native (CATH code 2kjwA00) and permuted sequence (CATH code 2kjwA00), MIR positions are clearly correlated to the presence of a regular secondary structure. The number of MIR is conserved for most of the strands but two: β2 and β4. β3 is located at the new N-terminal end, and β4 is located at the native C-terminal, but without any MIR in the permuted protein. In the combination of our methods, neither for the native (2kjvA00) nor for the permuted (2kjwA00), one finds a position being both MIR and H+2 in the β4 strand. In the native, the strand β4, formed by a succession of four hydrophobic amino acids, VMVV, is followed by FL at six positions downstream, i.e. a pair of hydrophobic residues. Since a contact between residues i and i+2 is not allowed, the presence of this FL pair may explain the formation of the six MIR at the C-terminal end of the protein, including β4. In the permuted sequence, the first hydrophobic residue following the β4 strand, is located further, at eight positions downstream. This difference may be sufficient to explain why no MIR is predicted in this strand for the permuted sequence.

## Conclusion

Prediction of the amino acids compulsory for the formation of the tertiary structure, not for the function, is the goal of this paper. As they are deeply involved in the interactions during the folding process, they are mainly hydrophobic and buried in the core of globular proteins. Two approaches developed by our two groups are combined to benefit of the synergy one can obtain from their joint use in order to decipher a very small proportion of key positions distributed throughout the sequences, typically of the order of 5%. On the one hand, a simulation originating from a single sequence produces a set of mainly hydrophobic amino acids compulsory for the folding to occur: the MIRs. On the other hand, the “fuzzy oil drop” (FOD) model compares the observed distribution of the hydrophobicity density of the amino acids to some ideal (theoretical) one, and thus produces a list of positions corresponding to the hydrophobic core of the globular protein. Both approaches support each other in order to hopefully reach an ideal amount of proportion of amino acids constituting the minimal nucleus, i.e. the deeply buried hydrophobic, compact, solvent hidden part, independently of their involvement in the process of folding itself. It is obvious, from the results on the two folds of this study, that comparison between experiments and predictions is in favour of the FOD relative to MIR, but one must remind that the fist one needs a structure while the second one does not. MIR prediction alone overestimates the number of intra chain contacts, due in part to the fact that the simulation is limited at the early step of the folding process. It thus presumably overestimates the number of short-range contacts, and underestimates the number of long-range contacts. Therefore, one may assume that MIR prediction is not sensitive to the symmetry of the domain, since the interaction length is only few tens of amino acids. In order to improve the quality of the MIR predictions, the idea of using some information from the structure has emerged and has been developed. One must also remember that experimental determination of the hydrophobic contact is not trivial [57] and the FOD model seems rather robust and evaluated among a large series of cases.

This procedure has been performed on a couple of folds: immunoglobulin-like and flavodoxin. With tens of structures available, each family is composed of distantly related sequences, therefore with certain diversity in the functions, but with a common fold. Application of the parametric hypergeometric law allows concluding that the positions both predicted as a MIR and assigned to the H+2 class is not the result of a hazardous choice, but rely on the physico-chemical properties of the folding nucleus both predicted from sequence and identified from 3D structure.

It is important to notice that the two methods, using different methodologies based on different folding states of the protein domains (early step of the folding, simulated from amino acid sequences by MIR method, and final state as resolved in the 3D structure and compared to an ideal Gaussian function by the FOD method), are in agreement, which is proven by significant intersection of their key predicted residues for both immunoglobulin-like and flavodoxin folds. In most cases, sources of any discrepancy may be explained on the basis of protein’s structural specificity [83]. The performance of joint prediction improves the fitness of the putative folding nucleus determination by significantly decreasing the number of positions capable of forming high levels of hydrophobic interactions. Comparison with reliable experimental data leads to reasonable quality of the prediction, which can nevertheless be further improved. There is a long running controversy in the literature concerning the conservation of the folding nucleus through proteins of common fold. According to the concept of Conservatism of conservatism proposed by the Shakhnovich’s group [37], the folding nucleus of a protein will be conserved in a protein family, because “interactions are more conserved than residues in protein evolution”. But for Jane Clarke, the Ig-like domain nucleus is centred on a common structural core, with some variation [46]. Our prediction model supports the second hypothesis more. In the case of Flav fold, widely described as an example of diffuse nucleus (with two cores in some cases), we also have been able by this method to evidence a very small number of positions of high number of contacts very well conserved throughout the proteins of this fold. To be clearer, conservation here must not be understood as a strict conservation, but rather among a degenerate alphabet aiming at the conservation of the hydrophobicity. Since we are using sets of proteins sharing a common fold, with sequence homologies in the so-called twilight zone, we do not focus on the questions of the uniqueness of the nucleus [32,84]. Rather, we do believe to be able to capture somehow the “conserved nucleus” among the members, which we assume to be some kind of signature for the fold.

Are these conclusions valid for other folds, regardless of the “conservation” of the nucleus? An unambiguous answer to this fundamental question not only implies analysis of one specimen of all the known folds, but also needs to consider some redundancy inside each fold. This paper is a first step in the proof of concept of the combination of the methods that must to be pursued in a systematic manner. To sum up, we can agree that we it is possible to analyse, either from the sequence or from the structure, the fine variations in the distribution of hydrophobicity, that—in given examples—can be considered a signature of the fold.

## Supporting Information

S1 Table Parameters of the hypergeometric distributions used to assess the statistical significance of the IR/FOD intertsecions for immunoglobulin-like (Ig) fold (A) and flavodoxin (Flav) fold (B), at the domain level. The p-value significance threshold is set to 1.3e-04 according to the Bonferroni correction (multiple hypothesis testing).
(DOCX)

S2 Table Experimental folding nucleus (A) (Ig fold) and core (B) (Flav fold): correspondence between multiple sequence alignment positions and PDB numbering. (DOCX)
