# Supplementary material for: Contribution to the Prediction of the Fold Code: Application to Immunoglobulin and Flavodoxin Cases
Source: PLoS One. 2015 Apr 27;10(4):e0125098. doi: 10.1371/journal.pone.0125098 (PMC4411048; doi:10.1371/journal.pone.0125098)
Supplement: S1 Table — The p-value significance threshold is set to 1.3e-04 according to the Bonferroni correction (multiple hypothesis testing). (DOCX) [file pone.0125098.s001.docx]

A

B

Table V. Parameters of the hypergeometric distributions used to assess the statistical significance of the IR/FOD intersections for immunoglobulin-like (Ig) fold (A) and flavodoxin (Flav) fold (B), at the domain level. The p-value significance threshold is set to 1.3e-04 according to the Bonferroni correction (multiple hypothesis testing).
