# Supplementary material for: Contribution to the Prediction of the Fold Code: Application to Immunoglobulin and Flavodoxin Cases
Source: PLoS One. 2015 Apr 27;10(4):e0125098. doi: 10.1371/journal.pone.0125098 (PMC4411048; doi:10.1371/journal.pone.0125098)
Supplement: S2 Table — (DOCX) [file pone.0125098.s002.docx]

A

| **Alignment position** | **Folding nucleus (PDB numbering)** | | | |
| --- | --- | --- | --- | --- |
|  | **1tenA00** | **1k85A00** | **1ttfA00** | **1titA00** |
| 129 | I821 | L578 |  |  |
| 131 |  |  |  | I23 |
| 166 |  |  | Y32 |  |
| 168 |  | V594 | I34 | W34 |
| 170 | Y837 |  | Y36 |  |
| 252 |  |  | F48 |  |
| 266 |  |  | V50 |  |
| **297** | **I860** | **I611** | **I59** | **L58** |
| 399 | V871 |  | I70 |  |
| 402 |  | V624 | V72 | F73 |

B

| **Alignment position** | **Core (PDB numbering)** | | |
| --- | --- | --- | --- |
|  | **1eayA00 core 1** | **1eayA00 core 2** | **1ftgA00** |
| 122 | L6 |  |  |
| 124 | F8 |  |  |
| 125 |  | L9 |  |
| **127** | **V10** |  | **L6** |
| 128 |  | V11 |  |
| 226 | M17 |  |  |
| 230 | V21 |  |  |
| 241 | L24 |  |  |
| 246 | L28 |  |  |
| 254 | F30 |  |  |
| 265 | V33 |  |  |
| 272 |  | A36 |  |
| 285 |  | D38 |  |
| 357 |  | A42 |  |
| 374 |  | L46 |  |
| 388 |  | Y51 |  |
| 394 | F53 |  |  |
| **395** |  | **V54** | **L50** |
| **396** | **I55** |  | **I51** |
| **397** |  | **S56** | **I52** |
| 399 |  | W58 |  |
| 457 |  |  | E61 |
| 465 |  | M60 |  |
| 470 |  |  | D65 |
| 471 |  |  | W66 |
| 473 |  |  | G68 |
| 474 |  | L68 |  |
| 476 |  | L69 |  |
| 477 |  |  | S71 |
| 479 |  | I72 |  |
| 483 |  |  | D75 |
| 494 |  | L81 |  |
| 499 |  | V83 |  |
| **500** | **L84** |  | **A84** |
| 501 |  | M85 |  |
| 502 | V86 |  |  |
| 555 |  |  | Q99 |
| 568 |  |  | I104 |
| 571 |  |  | E107 |
| 578 |  |  | I109 |
| 604 |  |  | V117 |
| 625 |  |  | T122 |
| 629 |  |  | D126 |
| 663 | F111 |  |  |
| 668 |  |  | V139 |
| 672 |  |  | L143 |
| 679 |  |  | D150 |
| 682 | A113 |  |  |
| 685 | L116 |  |  |
| 689 | L120 |  |  |
| 693 | I123 |  |  |
| 694 | F124 |  |  |
| 706 | M129 |  |  |

Table VI. Experimental folding nucleus (A) (Ig fold) and core (B) (Flav fold) : correspondance between multiple sequence alignment positions and PDB numbering.
